# Extracellular Vesicles in HTLV-1 Communication: The Story of an Invisible Messenger

**DOI:** 10.3390/v12121422

**Published:** 2020-12-10

**Authors:** Sarah Al Sharif, Daniel O. Pinto, Gifty A. Mensah, Fatemeh Dehbandi, Pooja Khatkar, Yuriy Kim, Heather Branscome, Fatah Kashanchi

**Affiliations:** Laboratory of Molecular Virology, George Mason University, Manassas, VA 20110, USA; salshar@gmu.edu (S.A.S.); dpinto1@gmu.edu (D.O.P.); gmensah2@gmu.edu (G.A.M.); fdehband@gmu.edu (F.D.); pkhatkar@masonlive.gmu.edu (P.K.); ykim78@gmu.edu (Y.K.); hbransco@gmu.edu (H.B.)

**Keywords:** HTLV-1, ATLL, HAM/TSP, extracellular vesicle, EVs, cell-cell contact

## Abstract

Human T-cell lymphotropic virus type 1 (HTLV-1) infects 5–10 million people worldwide and is the causative agent of adult T-cell leukemia/lymphoma (ATLL) and HTLV-1-associated myelopathy/tropical spastic paraparesis (HAM/TSP) as well as other inflammatory diseases. A major concern is that the most majority of individuals with HTLV-1 are asymptomatic carriers and that there is limited global attention by health care officials, setting up potential conditions for increased viral spread. HTLV-1 transmission occurs primarily through sexual intercourse, blood transfusion, intravenous drug usage, and breast feeding. Currently, there is no cure for HTLV-1 infection and only limited treatment options exist, such as class I interferons (IFN) and Zidovudine (AZT), with poor prognosis. Recently, small membrane-bound structures, known as extracellular vesicles (EVs), have received increased attention due to their potential to carry viral cargo (RNA and proteins) in multiple pathogenic infections (i.e., human immunodeficiency virus type I (HIV-1), Zika virus, and HTLV-1). In the case of HTLV-1, EVs isolated from the peripheral blood and cerebral spinal fluid (CSF) of HAM/TSP patients contained the viral transactivator protein Tax. Additionally, EVs derived from HTLV-1-infected cells (HTLV-1 EVs) promote functional effects such as cell aggregation which enhance viral spread. In this review, we present current knowledge surrounding EVs and their potential role as immune-modulating agents in cancer and other infectious diseases such as HTLV-1 and HIV-1. We discuss various features of EVs that make them prime targets for possible vehicles of future diagnostics and therapies.

## 1. Introduction

The human T-cell lymphotropic virus type 1 (HTLV-1) is an incurable blood-borne retrovirus that currently infects between 5 and 10 million people globally [1,2,3,4,5], where a majority may be lifelong asymptomatic carriers with the ability to unknowingly spread the virus [1,2]. It is likely that, due to limited universal screening, the true figure is greater as many cases are unreported from highly populated regions, such as China, India, Northwest Africa (i.e., the Arab Maghreb), and East Africa [6,7]. It is estimated that the actual global prevalence may be as high as 20 million worldwide [7,8,9]. Pockets of highly endemic regions exist in Japan, Australia, Iran, Jamaica, and Colombia [7], which exemplify localized regions from where the virus may spread. HTLV-1 is the causative agent of adult T-cell leukemia/lymphoma (ATLL) and HTLV-1-associated myelopathy/tropical spastic paraparesis (HAM/TSP), accounting for up to 5% and 3.8% of the infected population, respectively [3,10,11,12,13,14,15,16,17]. In addition, HTLV-1 is associated with other inflammatory diseases such as uveitis, rheumatic syndromes, bronchiectasis and infective dermatitis [18,19,20,21,22]. HTLV-1-infected patients are more susceptible to infectious diseases such as tuberculosis, disseminated *Strongyloides stercoralis*, and Norwegian scabies [23,24,25]. Therefore, it is possible that although many patients are asymptomatic, they may be more susceptible to development of secondary infections, such as the ones mentioned above.

### 1.1. Recent Trends in HTLV-1 Geographic Distribution

The data on HTLV-1 prevalence suggest that there may be: (1) new distribution trends, (2) increased viral transmission rates, and (3) pockets of high endemicity globally. The latter two could elucidate current epidemiological determinants of transmission and help to define prevention strategies of viral transmission worldwide. Recently, a significant increase (>40%) has been detected in the rates of HTLV-1 transmission in certain Indigenous populations of Queensland, Australia [26,27], suggesting a potential HTLV-1 re-emergence. This has drawn the attention of the scientific community. Interestingly, the re-emergence is not an isolated case since increased HTLV-1 transmission rates have also been reported around the world (as it is summarized in Table 1) [7].

For instance, HTLV-1 is geographically distributed with seroprevalence rates in Asia (high cases in southwestern isles of Japan), the Mashhad area of northeastern Iran [4,29], Caribbean islands (Jamaica) [9,37], and Central and South Africa [7,34]. A low prevalence of HTLV-1 infection has been reported in China, Iraq, Israel, Lebanon, Saudi Arabia, Turkey, Singapore, and South Korea [7]. Europe (France [9,35] and the United Kingdom [9,37]) and North America (United States) have a low prevalence, limited to groups of immigrants from endemic areas. HTLV-1 is also predominant in South and Central America (Colombia, Venezuela, Brazil, Guyana, Surinam, Panama, and Honduras) [39]. Collectively, the increases in HTLV-1 distribution in certain regions, such as in the Indigenous Australian population and Brazil, may be related to socioeconomic conditions (i.e., limited access to medical treatment, education, and the existence of stigmas associated with the infection) [41,42,43,44]. Additional research and education are needed to improve the knowledge about HTLV-1 pathogenesis and strategies to combat this devastating infection. 

### 1.2. HTLV-1 Transmission

The epidemiologic determinants of HTLV-1 transmission are similar to the human immunodeficiency virus type 1 (HIV-1). HTLV-1 is transmitted from mother to child (MTC), via sexual intercourse, needle sharing among drug users, and transfusion/transplantation of contaminated tissues [6,45]. The most efficient mode of transmission is via intravenous exposure with contaminated blood, 15–60%, followed by 20% efficiency of transmission from MTC through prolonged breastfeeding [46,47], and during peripartum, which occurs in approximately 5% of cases [48]. In certain populations, vertical transmission (i.e., MTC) is the most common route of HTLV-1 transmission, such as in the case of northern and central Australian Indigenous populations [10,43,49]. Similar trends have been observed recently in São Paulo, Brazil, where prevalence rates are currently increasing. MTC transmission occurs primarily via breastfeeding, with increased chances of transmission occurring in cases of prolonged breastfeeding [43]. Evidence suggests that breastfeeding for 12 months after birth has been associated with a 50% chance of MTC transmission, and infection rates of 14.2% out of 288 cases in Brazil [5,43]. As mentioned previously, low socioeconomic status is associated with higher risks of viral transmission, and in this case, primarily due to the lack of accessible alternatives to breastfeeding. Mothers with limited resources and knowledge about their HTLV-1 status can pass on HTLV-1 during birth or via breastfeeding. 

In Brazil, sexual transmission has been reported as the highest determinant of transmission [50], which mainly occurs from male to female [6,50]. The second most common mode of HTLV-1 transmission in Brazil is by intravenous drug use [44,51]. A recent study found elevated transmission rates of HTLV-1/2 in intravenous drug users (6.4%; *n* = 826) in certain Brazilian cities (i.e., State of Pará) [44]. Therefore, illicit drug users have a lifelong elevated risk of contracting HTLV-1. These data also indicate that in countries where HTLV-1 is a neglected virus, transmission rates have the potential to worsen, putting more people at risk [2]. Finally, blood transfusions are essential to standard care in hospital scenarios across the world. Screening for HTLV-1 in blood donors is required in only a handful of countries [2,35,40,52]. Unfortunately, many of the previously described countries have no HTLV-1 screening requirements and many others chose not to screen for the virus due to historically low prevalence [6,7]. Infection via blood transfusion has been associated with the development of HAM/TSP [52], whereas transmission via breastfeeding has been linked to ATLL [48]. Not surprisingly, vertical transmission of HTLV-1 has also been reported to affect, at higher rates, individuals of low socioeconomic background. Altogether, it is important to implement preventive and diagnostic public health policies to curb the risk of HTLV-1 transmission, particularly in low socioeconomic communities.

### 1.3. HTLV-1 Spread and Cell-Cell Contact

HTLV-1 spread within an infected individual may occur by clonal expansion of infected T cells or by viral transfer via cell-cell contact [53,54,55]. In contrast to HIV-1, free virions of HTLV-1 are not efficiently infectious as they are mostly undetectable in plasma, serum, or cell-free blood products [54]. Therefore, the primary modes of viral spread are via virological synapses (VS), viral biofilm (VB), cellular conduits (CC), and tunneling nanotubes (TNTs) [54,55,56]. Some of the molecular players that may allow cell-cell contact in HTLV-1 infection are the intercellular adhesion molecule 1 (ICAM-1), lymphocyte function-associated antigen (LFA-1), galectin-3, O-glycosylated surface receptors (CD43 and CD45), viral envelope glycoproteins (gp61/46), viral transactivator Tax, viral protein p8, carbohydrates, and components of the extracellular matrix (collagen, agrin) [54,56]. These molecules may promote cell contact and transmission of HTLV-1 virions from infected to uninfected neighboring cells, while evading early recognition by the immune system. Our recent publication, along with others in the field, has shown that ICAM-1 may be upregulated on the HTLV-1-infected cells, while its binding partner LFA-1 is commonly expressed by lymphocytes, allowing for potential cell-cell contact via formation of VS [57]. Similarly, CD43 and CD45 may be upregulated on the surface of infected cells, allowing for the formation of a VB and increasing the chances of cell-cell contact between uninfected and infected T cells. In neutralizing antibody assays, the viral proteins gp61/46 and Tax have been shown to be involved in enhanced cell-cell contact (uninfected T cells) and potentially, viral spread [57]. The viral protein p8 has been shown to participate in the formation of CC and TNTs by promoting formation of intracellular structures that project outward of the cell, into the extracellular environment, and establishing contact with adjacent cells [54,56]. Finally, other components of the extracellular matrix, such as carbohydrates, collagen, agrin, tetherin, and galectin-3, have also been shown to participate in cell-cell contact between infected and uninfected cells [54]. However, the extent of their involvement is not fully understood. Evidence suggests that they may play a supportive role in the adhesion observed in VS, VB, CC, and TNTs. Interestingly, our recent findings indicate that several of these cell-cell contact molecules may be bound to the surface or encapsulated within membrane-bound vesicles [57], elucidating a new avenue for molecular trafficking in HTLV-1 infection. 

Recently, membrane-bound vesicles (which may be formed intracellularly or may bud from the cellular membrane) have received increased attention due to their potential to carry cargo (i.e., proteins, RNA, and other cargo) and interact via autocrine, paracrine, and endocrine signaling. These membrane-bound structures are heterogeneous vesicles and altogether known as extracellular vesicles (EVs). EVs are mainly categorized based on their biogenesis, size, or cargo, and the field continues to define the distinct subpopulations. They are capable of packaging cargo and transporting it to the extracellular environment, and have been shown to play important roles in HTLV-1 pathogenesis [57,58]. EVs secreted by HTLV-1-infected cells have functional effects on recipient cells, including migration and agglutination, which in turn promote viral spread [57]. The role of EVs in HTLV-1 pathogenesis, and of viruses altogether, is more important now than ever before because it could hold the key to understanding the underlying mechanisms of viral infection.

## 2. Extracellular Vesicles (EVs)

### 2.1. EV Biogenesis

EVs is a term used for a heterogeneous group of double-layer phospholipid membrane vesicles released from virtually every cell [59]. EVs are classified according to their size, density, and associated cargo [60,61]. Based on size, EVs can be grouped into exomeres (<50 nm), exosomes (50–100 nm), microvesicles (MVs; 50–1000 nm), and apoptotic bodies (50–2000 nm) [62,63,64,65,66,67,68,69,70,71]. The membrane composition of EVs consists of lipid rafts, including ceramides, sphingolipids, cholesterol, and glycerophospholipids, as found in cellular membranes [72]. In the past, EVs were mainly considered waste carriers of the cell. However, it has now been shown that EVs carry a variety of cargo including proteins, nucleic acids, and lipids [60]. As a result, they mediate essential cellular processes such as cell-cell communication and immune functions. Interestingly, EV cargo reflect the originating cell rendering them potential biomarkers for clinical use [69]. EVs have also been discovered to affect the pathogenesis of several diseases and infections including cancer, HIV-1, and HTLV-1 [57,59]. Consequently, the interest in EVs including, but not limited to, their biogenesis, characterization, composition, and functions have significantly peaked over the years. Thus far, three EV biogenesis pathways have been established that may yield exosomes, MVs, and apoptotic bodies, and are the endosomal sorting complexes required for transport (ESCRT), transient plasma membrane budding, and membrane blebbing during apoptosis, respectively [59,69]. Clustering of cargo and membrane budding of exosomes can take place via either ESCRT-dependent or ESCRT-independent pathways. Exosomes are generated with the formation of intraluminal vesicles (ILVs) within multivesicular bodies (MVBs) or late endosomes which then fuses with the plasma membrane [73]. These released ILVs are then called exosomes. Tetraspanins such as CD9 and CD63 cluster proteins necessary for ILV formation serve as exosomal markers [73]. Four ESCRTs (ESCRTs 0, I, II, and III) are then localized at the budding site on the plasma membrane to facilitate the release of exosomes [73]. The ESCRT 0 and I subunits group ubiquitylated lipids and membrane-associated proteins on microdomains of MVBs and recruit ESCRT III (via ESCRT II) which performs and completes the budding process [69,73]. Certain ESCRT proteins have been shown to be necessary for the budding of enveloped viruses such as HIV-1 [74]. Therefore, the ESCRT pathway can be hijacked by viruses to release viral particles [59]. The ESCRT independent pathway calls for formation of ceramide, a sphingolipid which promotes the generation of membrane subdomains, resulting in a reshaping of the membrane curvature that induces budding of the MVB from the plasma membrane [69]. The tetraspanins CD9, CD63, CD81, and CD82 have also been shown to be involved in the ESCRT-independent pathway of exosome biogenesis [69]. These proteins form clusters with various transmembrane and cytosolic proteins potentially promoting the inward budding of the membrane and subsequent formation of an ILV [69]. The specific EV biogenetic pathway employed by cells significantly influences cargo type, which ultimately affects EV function.

EV biogenesis in HTLV-1 has not been widely studied. Additional research is needed to identify whether HTLV-1 EV biogenesis is mediated by ESCRT-dependent or -independent pathways. Use of siRNA to knockdown essential molecules involved in the aforementioned pathways and measuring the subsequent EV concentration could shed light on HTLV-1 EV biogenesis. It is important to reveal whether inhibiting one pathway of EV biogenesis could promote the other pathways (either positively or negatively) to form and regulate/release EVs. Additionally, evaluating changes in cargo packaging into EVs from HTLV-1-infected cells after knockdown of EV biogenesis pathways is worth examining. Such findings could be helpful in elucidating the role of infected EVs in HTLV-1 pathogenesis. 

### 2.2. EVs in Cell-Cell Communication

Under physiological or pathological conditions, almost all cells secrete EVs that can be detected in biological fluids, such as blood, urine, sputum, breast milk, amniotic fluid, cerebrospinal fluid (CSF), and semen [75,76,77,78,79,80,81,82,83]. These EVs contain proteins, lipids, DNA, and mRNA, miRNA, long non-coding RNA, or genetic material of pathogens (e.g., viruses and bacteria) [57,64,84,85,86,87,88]. Moreover, EVs can carry different types of cellular molecules, such as adhesion molecules, membrane proteins trafficking molecules, cytoskeleton proteins, heat-shock proteins, cytoplasmic enzymes, signal transduction proteins, cytokines, chemokines, proteinases, apoptotic cells markers, and pro- or anti-apoptosis factors [89]. The diversity of EV composition, as determined by cargo bound to the surface or encapsulated within, yields potential differences in their functional properties and effects on recipient cells.

#### 2.2.1. EV Cargo in Viral Infection

There are numerous surface molecules in EVs that are able to bind many cell receptors, making possible the interaction between cells and exchange of cargo [90]. Additionally, normal cellular functions, such as phagocytosis in many immune cell types, allow EV uptake by the target cell [91] or direct fusion with the plasma membrane (i.e., clathrin-mediated endocytosis) [92,93]. Once inside, the cargo in EVs may regulate cellular functions in recipient cells, such as immune responses [94] and inflammation [95]. EVs may enhance the spread of infection via the transfer viral cargo, helping pathogens to escape immune detection, while inducing apoptosis of recipient immune cells [96]. It has been shown that HIV-1 infection promotes the release of EVs containing viral molecules and host components from the infected cells (e.g., monocytes, macrophages, dendritic cells, and T cells) [87,97,98], pointing to the essential role of EVs in viral spread. For instance, EVs derived from an uninfected T-cell line (i.e., CEM cells) were shown to reverse latency in HIV-1-infected cells in a process mediated by EV-associated to a non-receptor tyrosine kinase (c-Src) [99,100]. This kinase is involved in NF-kB signaling pathways, involved in cellular proliferation, differentiation, motility, and angiogenesis. The c-Src in EVs may also be able to regulate such cellular processes. In addition to the functional effects on activation of NF-kB in recipient cells, EVs from uninfected cells may also facilitate viral entry. EVs containing mucin proteins (TIM-4) bind to phosphatidylserine (PS) on the virion surface and T cell immunoglobulin which facilitate cell-cell contact and HIV-1 infection in the recipient T-cells [101]. Additionally, tetraspanins (i.e., CD9 and CD81) in EV membranes have been found to aid HIV-1 entry in T-cell and macrophage cell cultures. Moreover, viral entry into the recipient cells could be restricted by use of antibodies against TIM-4, CD9, and CD81 in EVs [101,102]. Therefore, EVs targeting recipient cells, alongside virions, such as HIV-1, may synergize to increase efficiency of viral entry. Collectively, the roles of EVs, along with viral particles, in target cells need to be further investigated, and close attention should be given to the cargo which play a dynamic role in disease pathogenesis.

Another key example of the EV roles during infection is during infection by Zika virus (ZIKV). ZIKV is an emerging mosquito-transmitted virus that causes microcephaly and other congenital malformations in the infant. EVs isolated from ZIKV-infected mosquito cells have been shown to deliver viral RNA and ZIKV-E protein into the main target cells (i.e., monocytes and endothelial vascular cells) for ZIKV infection. These EVs stimulate the differentiation of naïve monocytes, the production of TNF-α mRNA and the damage of endothelial vascular cells. These changes mediate the inflammation in human cells and could participate in ZIKV pathogenesis [103]. A recent study has shown that engineered mammalian EVs, that carry interferon-induced transmembrane protein 3 (IFITM3), were effective in reducing the viral titers in pregnant mice. These EVs were also found to pass through the placental barrier and protect the fetus from ZIKV infection. These findings indicate that EVs containing IFITM3 can be used as treatment to minimize ZIKV infection during pregnancy [104]. 

In HTLV-1 infection, two viral regulatory proteins, Tax and HTLV-1 basic zipper protein (HBZ), have crucial roles in leukemogenesis of ATLL [105,106]. The oncoprotein Tax is attributed as the primary cause of the abnormal proliferation of HTLV-1-infected cells, as cells normally require Interleukin 2 (IL-2) for proliferation. However, in later stages of infection, a correlation has been observed between higher levels of Tax and IL-2-independent proliferation [107]. Moreover, HBZ also stimulates cell survival and proliferation of infected cells in ATLL patients. Yet, the mechanisms of progression from HTLV-1 infection to ATLL are still not fully understood. Our recent work has shown that HTLV-1-infected cells release EVs containing viral RNA and proteins (gp61/Tax/HBZ) and, interestingly, these EVs are not infectious [57]. However, data also suggest that EVs secreted from infected cells may enhance cell-cell contact between infected cells and recipient uninfected cells (T cells, myeloid cells, and peripheral blood mononuclear cells (PBMCs)) [57]. This increased cell-cell contact, along with other not yet fully understood mechanisms, promotes viral spread into uninfected cells, as evidenced by significantly increased RNA levels in cell lines and PBMCs. This was also true for viral spread in vivo since elevated RNA and proviral DNA levels were detected in a NOD/Shi-scid/IL-2Rγc ^null^ (NOG) humanized (hu-NOG) mouse model treated with EVs [57]. Understanding the mechanisms by which these EVs promote increased cell-cell contact, and subsequently viral spread, may be important to further elucidate the HTLV-1 pathogenesis and development of diseases such as ATLL and HAM/TSP.

It is known in the field that HTLV-1 transmission occurs primarily via cell-cell contact, and that some of the molecular players involved are cell surface receptors and other cellular components which act at the interface between cells. Since EVs may carry several of these receptors, as well as viral proteins encapsulated within, they may be essential players in these modes of cell-cell contact [57,58]. Experiments using neutralizing antibodies against cellular surface receptors (e.g., CD45, ICAM-1, VCAM-1, and LFA-1) to hinder cell-cell contact showed the potential importance of CD45 (or its receptor CD43) and ICAM-1 in facilitating the viral spread through mechanisms known as VB formation or VS, respectfully [57]. Indeed, the presence of CD45 and ICAM-1 in EVs has been confirmed, via Western blotting, to be released from HTLV-1-infected cells. Additionally, increases in the level of these cellular surface receptors could promote contact between infected and uninfected cells to support viral spreading [57]. As mentioned previously, data from hu-NOG mice treated with HTLV-1 EVs, followed by HTLV-1 infection, showed that viral RNA synthesis increased in mice exposed to HTLV-1 EVs in contrast to mice exposed to EVs obtained from uninfected cells [57]. Moreover, an increase in proviral DNA levels in mice tissue (i.e., blood, lung, spleen, liver, and brain) was observed in mice that received HTLV-1 EVs [57], suggesting the potential curial role of HTLV-1 EVs in viral spread and disease progression. To further support these findings, we have also isolated EVs from HAM/TSP patient PBMCs and CSF, which contained Tax, with functional effects in Tax-specific cytotoxic T-cell lysis [82]. This suggests the potential negative effects that EVs from HTLV-1 infected cells may have in the central nervous system (CNS). Interestingly, we have recently observed that EVs from HTLV-1 affect cells in an angiogenesis assay, which represents the vascular-like structures presenting the blood brain barrier (BBB). The assay consists the use of EVs from HTLV-1-infected cells to treat a co-culture of aortic endothelial cells and mesenchymal stem cells (MSCs) [58]. EVs decreased the overall number of vascular structures in the assay, suggesting a negative effect towards angiogenesis [58]. Other in the field have also reported the functional effects of EVs from EVs from HTLV-1-infected cells on MSCs [108]. These EVs contained Tax, vascular endothelial growth factor (VEGF), and leukemia-related miRNAs that promote changes in MSC properties to support tumor growth [108]. Additionally, the proinflammatory cytokines, IFN-γ and TNF-α, were detected in healthy PBMCs after exposure to EVs isolated from HTLV-1-infected cells. This finding indicates that EVs containing Tax mediate the release of these two cytokines and the activation of T helper _1_ (Th1) immune response in the treated PBMCs [109]. This finding could explain the inflammation which is predominantly seen in HAM/TSP patients with Th1 phenotype [110]. Altogether, these data validate the clinical relevance of EVs in HTLV-1-related diseases. 

It is known that EVs are heterogenous in nature, primarily due to their size and associated cargo. Therefore, it is important to fully evaluate the composition of EVs isolated from HTLV-1. Proteomic analysis of plasma EVs from HTLV-1 asymptomatic carriers, as well as in HAM/TSP patients, shows that EVs are largely found in high concentrations (100–200 × 10^9^ particles/mL), small in size (around 70 nm), and enriched with markers of metabolic and mitochondrial stress [111], suggesting a potential function of these smaller EVs in inflammation, cellular stress and tissue damage, and cancer development. 

#### 2.2.2. EV Cargo in Cancer

It has been reported that EV biogenesis is de-regulated in cancer cells, and that these cancer cells tend to release a higher amount of EVs compared to healthy cells [112,113]. Many publications have extensively shown that EVs derived from cancers contain oncogenic molecules which remarkably regulate the biological events of their recipient cells. For instance, cancer-derived EVs can change local and systemic cellular microenvironments including supporting tumor growth [114], therapy resistance [115], and metastasis [67,116]. In the cancer field, EVs have been studied because of their roles in modulating the immune system, as well as their potential functions in promoting tumor metastasis [116]. It has been found that cytokines (e.g., IL-6 and tumor growth factor-β1 (TGF-β1)) released from cancer cells [117]. EVs can interfere with the activation of immune cells [117]. A recent significant publication showed that cytokines may be found on the surface of EVs or can be packaged inside EVs [118]. These cytokines enclosed in EVs could potentially participate in disease progression. Future studies are needed to assess the biological roles of cytokines in EVs regardless of the disease. An advantage of utilizing EVs to carry cytokines could be to protect them from degradation and deliver them to cells at distant sites to stimulate specific responses such as priming the environment for infection.

Tumor EVs may be involved in the process of cell transformation by protecting cancer cells from being destroyed by the host immune cells. Tumor EVs were observed to prevent the response of cytotoxic effector cells (i.e., CD8+T lymphocytes and natural killer (NK) cells) to IL-2 stimulation which is crucial for their proliferation [119]. On the other hand, tumor EVs inhibited the host immune reaction by inducing the production of immunosuppressive regulatory T cells via increased FoxP3 [119]. The localization of TGF-β1 on the surface of these tumor EVs could potentially provide additional antiproliferative effects [119]. A recent study reported that EVs isolated from pancreatic cancer cells were only able to initiate malignant cell transformation of normal recipient cells by creating random mutations and along with other factors have been observed to cause cancer in vivo [120]. Prostate cancer cell-derived EVs have been shown to carry tumorigenic molecules (e.g., *ras* transcripts (H-*ras* and K-*ras*), Rab proteins (Rab1a, Rab1b, and Rab11a), and oncogenic miRNAs (miR-125b, miR-130b, and miR-155) that block tumor suppressors such as Lats2 and PDCD4 to allow transformation of adipose-derived stem cells in primary as well as metastatic sites in cancer patients, eventually promoting tumor clonal expansion [121]. Taken together, these data point to cancer EVs as essential regulators of cancer progression. Understanding whether such EVs have a role in cell transformation of non-tumorigenic cells could aid in designing therapies to target tumor EVs and/or their cargo. 

It is known that cancer cells are self-sufficient in growth signals that sustain their growth and survival [122,123]. As previously mentioned, EVs contain cytokines located either inside or on the surface that may mediate disease progression. A recent study has shown that chronic myeloid leukemia (CML) cells release EVs (i.e., exosomes) containing TGF-β1. These EVs are taken up by the same cells (i.e., in autocrine feedback loop), and are able to affect their growth and survival in a time- and concentration-dependent manner [114]. In addition, Ren et al. 2019 found that EVs derived from colon cancer cells accelerated cell division, which in turn contributed to disease progression [124]. Another study examining gastric cancer EVs also found increased cell proliferation in a time and concentration manner through activation of Akt and ERK, and PI3k pathways [125]. In all, based on the progress and consistent data from various sources in the field of cancer EV pointing to the role of EVs as key participants in the progression of disease, we may be able to extrapolate important information to better understand HTLV-1 pathogenesis related to ATLL.

## 3. EVs in HTLV-1-Related Diseases

EVs are actively involved in cell-cell communication and mediate disease progression [62,64,66]. HTLV-1 uses EVs for incorporating their viral molecules for intracellular communication [57,82,88]. HTLV-1 is known to be associated with ATLL [13] and inflammatory disease states including HAM/TSP [18], infective dermatitis (IDH) [126], pulmonary diseases (bronchiectasis [127], alveolitis [128]), and HTLV-1-associated uveitis (HU) [21,129]. In this section, we will explore the role of EVs in each of these HTLV-1-associated diseases.

### 3.1. EVs in ATLL

ATLL was first recognized as a new disease in 1977 in Kyoto, Japan [130]. Subsequently, it was discovered that HTLV-1 is the causative agent of ATLL in 3 to 5% of HTLV-1 cases [3,13,131]. ATLL clinical indicators include leukemic manifestations, generalized lymphadenopathy, hepatomegaly, splenomegaly, skin involvement, hypercalcemia, and infiltration of other organs (e.g., CNS and gastrointestinal tract). ATLL is classified into four clinical types (i.e., smoldering, chronic, lymphoma, and acute) based on site of leukemic cell infiltration, degree of leukemic symptoms, lactate dehydrogenase level, and hypercalcemia [132]. ATLL cells have a mature helper T-cell phenotype (CD3^+^, CD4^+^, CD8^−^), and regulatory T cells ((CD25^+^CCR4^+^FoxP3^+^ (50% of patients express FoxP3^+^)) [133]. The mechanisms of HTLV-1 infection mediating ATLL development are not fully elucidated. The abnormality of immune responses to HTLV-1-infection are mediated through production of different cytokine networks and may participate in ATLL development. Accumulation of immunosuppressive cytokines (i.e., IL-10 and TGF-β), released by regulatory T cells, induce the proliferation of transformed HTLV-1-infected cells [133]. IL-10 production is mediated by HTLV-1 Tax and HBZ [134]. While Tax induces TGF-β production, it also inhibits TGF-β/Smad signaling and FoxP3 expression, making HTLV-1-infected cells resistant to TGF-β-mediated growth inhibition [135]. In ATLL cells, HBZ promotes TGF-β/Smad signaling and increases the expression of FoxP3 that generate more regulatory T cells from HTLV-1-infected T cells. However, HBZ promotes the loss of FoxP3 function [136]. These findings may contribute to viral persistence. Sawada et al. 2017 showed that autocrine or paracrine feedback loops of IL-10 mediated by either the HTLV-1-infected cell and/or the neighboring cells promote the lymphoproliferative features of HTLV-1, subsequently leading to leukemogenesis [137]. Additionally, the uncontrolled proliferation of HTLV-1-infected cells observed in ATLL patients could be due to the low activity of Tax-specific cytotoxic T lymphocytes [138]. Furthermore, it has been shown that Tax enhances uncontrolled replication of T cells and cellular genes (e.g., IL-2/IL-2R), disrupts DNA repair mechanisms causing genetic instability, inhibits apoptosis of infected cells, and interferes with cell cycle check points [139,140]. Based on these results, it was concluded that HTLV-1 Tax promotes oncogenesis and the subsequent development of ATLL. 

**Figure 1:**
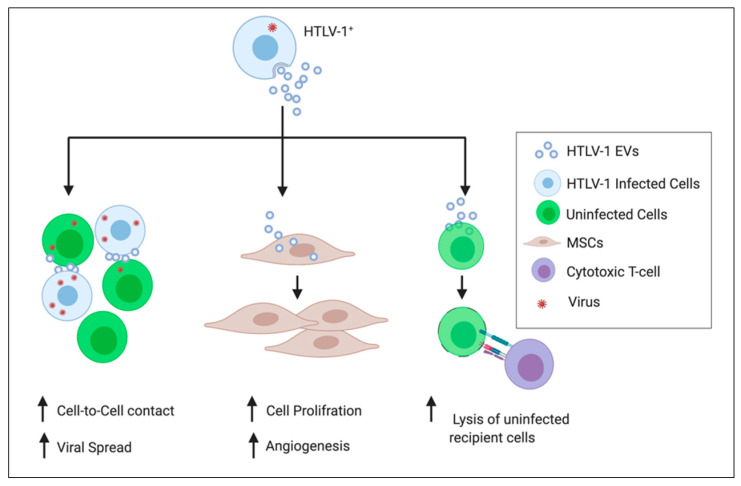
The effect of HTLV-1 EVs on uninfected recipient cells. HTLV-1 EVs could enhance cell-cell contact between HTLV-1-infected cells and uninfected T cells, thereby increasing viral spread. HTLV-1 EVs supports cancer progression by inducing the proliferation and angiogenesis of mesenchymal stem cells (MSCs). Additionally, HTLV-1 EVs prompt the lysis of uninfected target cells by cytotoxic T-cells, potentially resulting in chronic inflammation. Figure adapted from [57,108].

Recent publications have revealed that EVs have a key involvement in viral pathogenesis and that they can manipulate host immune responses to promote viral infection [141]. In the context of HTLV-1 and EVs, a novel significant study by Jaworski et al. 2014 demonstrated that HTLV-1-infected T cells release EVs containing viral Tax protein [88]. This study showed that the EVs secreted from HTLV-1-infected cells may protect uninfected cells from Fas-mediated apoptosis by increasing cFLIP and NF-κB activity, enhancing survival of IL-2-dependent CTLL-2 cells through activation of AKT [88]. Moreover, HTLV-1 EVs were found to enhance survival of PBMCs in the absence of exogenous growth stimulation [88]. Likewise, a recent study showed that HTLV-1 EVs promote cell-cell contact and amplify viral spread, suggesting the potential role of EVs in HTLV-1 pathogenesis (Figure 1) [57]. This study showed an increase in CEM T cells agglutination post-HTLV-1 EVs treatment with the most prominent effect noticed following HTLV-1/IR EVs treatment. Moreover, viral RNA levels (i.e., *tax, env,* and *hbz*) were induced after incubation of uninfected CEM T cells with HTLV-1/IR EVs and irradiated HTLV-1 cells for 5 days [57], signifying viral spread. 

The EV-mediated disease mechanism appears to be analogous with hormone signaling pathways. In endocrine signaling, an extracellular mediator released from one cell acts on a distant cell [142]. Similarly, it was shown that melanoma cell EVs travel to Sentinel Lymph Nodes and condition the microenvironment in the lymph Node to facilitate lymphatic metastasis of cancer cells [143]. EVs isolated from an ATLL cell line were found to promote alteration in MSCs providing a suitable microenvironment for leukemia [108]. MSCs post-treatment with ATLL EVs showed changes in MSCs morphology, enhanced their proliferation, gene expression of migration (matrix metalloproteinase-9 (MMP-9) and chemokine receptor type 4 (CXCR-4)) and angiogenesis (VEGF) markers. These changes may be mediated by cargoes within ATLL EVs (i.e., Tax, miR-21, miR-155, and vascular endothelial growth factor) (Figure 1) [108]. In regard to the paracrine mechanism of action by EVs, several studies have shown data supporting the hypothesis that EVs from donor cells act and elicit functional changes on their neighboring cells [57,66,99] 

In autocrine signaling, an extracellular mediator secreted by a cell binds to the same cell receptors to initiate signal transduction [142]. While there is growing data on the role of exosomes acting in a paracrine fashion within the tumor microenvironment, little is known about their role in affecting the growth and survival of the releasing cells via an autocrine mechanism. In terms of HTLV-1, it has been shown that Tax induces an autocrine loop through IL-17RB-NF-κB signaling which was found to be essential for HTLV-1 leukemogenesis [144]. It might be possible that Tax initiating the IL-17RB-NF-κB feed-forward autocrine loop is encapsulated in EVs and helps the self-renewal of infected cells via a regulatory mechanism. 

### 3.2. EVs in HAM/TSP

Approximately 0.25–3.8% of HTLV-1-positive patients develop HAM/TSP. HAM/TSP, a clinical neuroinflammatory disease, is characterized by chronic spinal cord inflammation and progressive myelopathic symptoms (e.g., spastic paraparesis, dysuria, and lower limb weakness) [145]. Due to the lack of effective treatments for HAM/TSP, patients become disabled after a year of illness [146]. High levels of HTLV-1 proviral load were detected in PBMCs of HAM/TSP patients (i.e., 16-fold greater) compared to asymptomatic carriers [147]. Similar results were observed in the polyclonal proliferation of HTLV-1-infected T cells [148]. Additionally, it has been reported that HAM/TSP patients display spontaneous lymphoproliferation [149]. Two cytokines, IL-2 and IL-15, can mediate HAM/TSP pathogenesis. Tax protein induces the expression of IL-2 and IL-15 and their receptors causing activation of lymphocytes in HAM/TSP patients. In an ex vivo setting, spontaneous lymphoproliferation was observed without exogenous stimulation (IL-2 and IL-15) [150,151], suggesting an autocrine IL-2 proliferation loop. Moreover, IL-15 plays a role in maintaining the survival of CD8^+^ T cells (especially, memory CD8^+^ T cells) [152]. Besides the increased level of proviral load, persistently activated immune responses against HTLV-1-infected cells could potentially be the main cause of the increased inflammation seen in individuals suffering from HAM/TSP. It is thought that CD4^+^ HTLV-1-infected T cells and activated IFN-γ T cells have access to the CNS. Tax induces the production of IFN-γ from T cells [15]. IFN-γ induces astrocytes to produce CXCL10 that attracts both uninfected and HTLV-1-infected CD4^+^ and CD8^+^ CXCR3^+^ T cells into the CNS [153], causing more damage in the CNS via repeating cycles between IFN-γ production, astrocytes secretion, and the infiltration CXCR3^+^ T cells [153]. Additionally, HTLV-I-infected T cells secrete MIP-1α within the CNS, allowing activated monocytes and T lymphocytes ((MIP-1α receptors (CCR1^+^)) to access the sites of inflammation in the CNS [154]. Macrophages produce MCP-1 within the CNS that may stimulate the penetration of peripheral blood monocytes (CCR2^+^) into the CNS during HTLV-1-infection [154]. MMP-2 and MMP-9 are found to be highly expressed in CSF and CNS. This may promote the destruction of the integrity of the BBB in HAM/TSP patients [155]. Moreover, Tax has been linked to the enhanced expression of cell adhesion molecules (e.g., ICAM-1, VCAM-1, CADM1/TSLC1, and ALCAM/CD166) that allow HTLV-1-infected T cells to cross the BBB endothelium [155]. In the CNS, macrophages, astrocytes, and microglia present at the site of inflammation produce inflammatory cytokines such as IL-1β, TNF-α, and IL-6. HTLV-1 can infect Tregs T cells (CD4^+^CD25^+^ T-cells) and result in loss of suppressive function [156]. Uninfected CD4^+^CD25^+^ T cells transduced with Tax showed a reduction in the mRNA level of *FoxP3* as well as loss of suppressive function [156], leading to a weakened immune response. CD4^+^ CD25^+^ T cells isolated from HAM/TSP patients showed an increased level of HTLV-1 proviral load that promotes the secretion of INF-γ and other cytokines [157]. CD4^+^CD25^+^ T cells induce the proliferation of HTLV-1 Tax-specific CD8^+^ T cells [157] that may contribute to the pathogenesis of HTLV-1 in HAM/TSP patients. A high presence of Tax-specific CD8^+^ T cells as well as anti-HTLV-1 antibody, altogether, results in chronic inflammatory responses that cause neural and spinal cord damage [15]. 

EVs may contribute to the pathogenesis of HTLV-1 in HAM/TSP. EVs (i.e., exosomes) isolated from HTLV-1-infected cell lines have been shown to contain Tax protein, viral mRNA transcripts (i.e., Tax, HBZ, and Env) as well as proinflammatory mediators (i.e., Granulocyte-Macrophage Colony Stimulating Factor (GM-CSF) and IL-6) [88]. It has been shown that Tax protein was present in EVs obtained from the CSF of HAM/TSP patients. However, viral particles are not usually detected in the CSF supernatant [82]. The same study showed that HAM/TSP CD4^+^CD25^+^ T cells can release EVs carrying HTLV-1 Tax that are immunogenic, and can promote the lysis of uninfected recipient cells by HTLV-1-specific cytotoxic T cells (Figure 1) [82]. These findings imply that EVs deliver HTLV-1 molecules and may contribute to triggering an immune response in the CNS, subsequently causing neurodegeneration in HAM/TSP. In our recent published study, we showed that HTLV-1 EVs increase cell-cell contact in uninfected T cells potentially facilitating viral transmission in vitro [57]. We found that ionizing radiation (IR) treatment induced the release of EVs from HTLV-1-infected cells as well as Tax packaging into EVs [57]. Additionally, HTLV-1 DNA levels in the brain of infected animals were significantly higher in mice that were treated with HTLV-1 EVs compared to mice treated with uninfected control EVs. These results suggest that HTLV-1 EVs could participate in viral spread across the BBB and may contribute in the development of HAM/TSP. Further investigation is needed to understand how EVs enhance cell-cell contact for viral spread. Such findings could lead to elucidating the mechanisms of HTLV-1 pathogenesis and development of HAM/TSP. 

Finally, there is very little information on whether Tax within EVs could be involved in the induction of proinflammatory cytokines in recipient CNS cells (i.e., macrophages, astrocytes, and microglia); and whether HTLV-1 EVs containing cytokines play a role in HTLV-1-associated HAM/TSP. Moreover, it is not clear whether HTLV-1 EVs could participate in the spontaneous lymphoproliferation observed in HAM/TSP patients. To better understand the comprehensive mechanism of EV-mediated inflammation in CNS, a detailed study oriented towards EVs and HAM/TSP performed from multiple patient sample cohorts could address these potentially significant issues. 

### 3.3. EVs in Infective Dermatitis

Infective dermatitis associated with HTLV-1 (IDH) is a rare but important HTLV-1-related disease due to increases in the geographical distribution ranging from the Caribbean (i.e., Jamaica) to South America (i.e., Brazil) and Africa (i.e., Senegal) [158]. IDH is mainly observed in young children of approximately 18 months of age who contracted HTLV-1 via vertical transmission [158,159]. IDH can be diagnosed by detection of anti-HTLV-1 antibodies against viral proteins (p24, p19, gp46-1, and gp21) in blood or CSF samples [160]. Due to the development of the immune system, IDH disappears when patients reach adolescence [158,159]. IDH turns into a chronic exudative eczematous eruption affecting the scalp, earlobes, neck, axillae, and inguinal and periumbilical skin once the patients discontinue antibiotics [161]. The first case of IDH was reported in 1966 in Jamaica [162]. In 2011, other HTLV-1 endemic regions (e.g., Senegal and Brazil) reported many new cases of IDH [158], providing more evidence of the association between increased transmission of HTLV-1 and IDH. A first landmark study in 1990 showed that IDH is an early marker of HTLV-1 [20]. This was later confirmed by a case-control study performed by the same group [163]. Along the same line, several studies showed individuals that developed IDH early in life had higher chances of developing ATLL or HAM/TSP (54%) later in life [126,164,165,166]. However, IDH patients may more often progress to HAM/TSP earlier [167] due to humoral anti-HTLV-1 responses and elevated proviral load [168]. IDH is also characterized by an increase in CD4^+^ and CD8^+^, a CD4^+^/CD8^+^ ratio of 1:73, and hyperexpression of IgD or IgE [161,163]. Most of the CD8^+^ T lymphocytes in IDH patients have not been found to have cytotoxic effect (i.e., lack of granzyme B granules and perforin) [167], and therefore the patients may be in an immunosuppressed state. Elevated IgE levels in IDH patients potentially allow them to become more susceptible to superinfection with *Staphylococcus aureus* (*S. aureus*) and *beta-haemolytic streptococci* (*BHS*) [169]. The mechanisms of this immunosuppression in IDH patients could be a result of HTLV-1 viral spread stemming from the limited proliferation of naïve T cells in the thymus and increased expression of programmed death (PD-1) and its ligand, ultimately leading to reduced T cell immune responses [169]. Moreover, regulatory T cells expressing FoxP3 were abundant in tested skin biopsies obtained from IDH patients, promoting localized immunosuppression (i.e., inactive CD8^+^ T cells) and tumorigenesis [169]. Additionally, the viral protein Tax has been shown to elicit production of proinflammatory cytokines (i.e., IL-1, IL-6, and TNF-α) [169,170]. Therefore, the proximity of these cell types to lesions in the cutaneous layers of the epidermis, as well as the high levels of Tax, could explain the inflammation seen in the cutaneous lesion in IDH patients. 

However, the role of EVs in infective dermatitis largely remains unexplored. A study showed that 5 out of 35 patients with AD and all 50 patients with IDH had been diagnosed with HTLV-1, and both groups had *S. aureus* and/or *BHS* [171]. Another recent study has shown the involvement of EVs in dermatitis. It was observed that EV-associated α-Hemolysin secreted by *S. aureus* induced keratinocyte death, skin barrier disruption and inflammation (release IL-1β, IL-6, IL-8, and MIP-1α)—the key phenotypes associated with atopic dermatitis (AD) [172,173,174]. Additionally, it was found that there was an increase in the pro-inflammatory mediators, including IL-6, thymic stromal lymphopoietin (TSLP), MIP-1α, and eotaxin, in mouse dermal fibroblasts cells exposed to *S. aureus* EVs [175]. Additionally, mast cells and eosinophils were accumulated and established the epidermal thickness of mice skin exposed to *S. aureus* EVs [175]. This was explained by an increase in the cutaneous secretion of IL-4, IL-5, IFN- γ, and IL-17 from mixed Th1-/Th17-/Th2 type cells [175]. Altogether, these findings suggest that *S. aureus* EVs mediate pathogenesis of AD and could be used as a therapeutic target to reduce AD spread. 

A recent study performed in a mouse model showed that adipose tissue-derived mesenchymal stem cell-derived exosomes (ASC exosomes) successfully decreased systemic inflammation and ameliorated AD symptoms [176]. Ceramide is the main crucial lipid bilayer component of stratum corneum of the epidermis and has a key role in water control retention and permeability functions. A reduction in total lipid and ceramides that alter the integrity of the epidermal barrier have been observed in AD [177]. ASC exosomes were found to recover epidermal permeability barrier in AD via induction of ceramide formation and suppression of immune responses [178]. The above findings indicate the potential use of ASC exosomes to treat AD. 

In addition to the aforementioned works on IDH and AD, several studies have shown the role of EVs in HTLV-1 pathogenesis. EVs isolated from cell lines, PBMCs, and CSF samples have been shown to contain Tax protein. Although not proven, but HTLV-1 EVs (via their cytokines and viral proteins/nucleic acids cargoes) could affect IDH pathogenesis by inactivating the cytotoxic functions of CD8^+^ T cells. Elucidating the mechanism underlying how HTLV-1 EV-associated cytokines (whether surface-bound or encapsulated) mediate the mechanism of CD8^+^ T cell inactivation and IDH pathogenesis could reveal possible mechanisms involved in IDH progression.

### 3.4. EVs in Pulmonary Diseases

A recent case-controlled study performed in central Australia showed that bronchiectasis played a role in an increase in the death rate among HTLV-1-positive Indigenous Australian adults [27]. High viral load (i.e., pVL > 1000 copies/10^5^ peripheral blood leukocytes), virus subtype (i.e., HTLV1c subtype), genetics, and limited cytotoxic T lymphocyte effect were linked to the severity of lung disease [27]. High-resolution computed tomography of the chest (CT) images taken from Japanese patients with HTLV-1 showed lung parenchymal abnormalities (i.e., mainly thickening of bronchovascular bundles and bronchiectasis); and lung biopsies displayed lymphocytic infiltration along respiratory bronchioles and bronchovascular bundles [179]. A series of studies has shown the relationship between HTLV-1 and pulmonary diseases. Specifically, HAM/TSP patients displayed lymphocytic inflammation [180,181,182] and changes in CT scan images [183,184,185]. Severe lung damage is predominantly observed in HTLV-1-associated inflammatory diseases such as HAM/TSP and uveitis due to the presence of high CD4^+^CD25^+^ T lymphocytes, release of cytokines (i.e., IL-2, IL-12, and IFN-γ), inflammatory chemokines (i.e., MIP-1α and IP-10), and expression of ICAM-1 in the bronchioalveolar lavage fluid (BALF) [127,186]. The presence of lymphocytes in large quantities have shown to be positively correlated with high levels of *FoxP3* mRNA in the BALF of patients with HTLV-1-related lung diseases [187]. Moreover, one of the findings showed increased expression of the viral mRNA (*Tax* and *HBZ*) in the BALF cells of patients with HTLV-1 [187]. Tax is responsible for inducing inflammatory chemokines in order to activate and attract inflammatory cells into the lungs [128]. Taken together, it can be concluded that HTLV-1 infection has a positive correlation with the level of inflammation seen in patients with alveolitis and bronchiectasis. 

Regarding the role of EVs in pulmonary disease, studies have shown the role of EVs in other pulmonary diseases such as chronic obstructive pulmonary disease (COPD), pulmonary hypertension (PH), and Asthma, among others. A series of studies have shown the role of specific miRNA containing exosomes in pulmonary diseases such as increased concentration of miR-210 [188] and EV-miR-21 in COPD patients [189] where former miRNA controls the autophagy functions and differentiation of myofibroblasts when present in the bronchial epithelial cell-derived EVs. Likewise, exosomes enriched in miR-143, produced by pulmonary arterial smooth muscle cells (PASMCs), induce the migration and angiogenesis of pulmonary arterial endothelial cells (PAECs) and contribute to the pathogenesis of PH [190]. Similarly, increased levels of EV-associated miRNAs such as miR-629-3p, miR-223-3p, miR-12-3p, miR-155 miR-125b, miR-16, miR-299-5p, miR-126, miR-206, and miR-133b have been reported in severe asthmatic patients [191,192,193,194]. Interestingly, some studies have reported EVs associated proteins, which plays a major role in different signaling pathways where 15-LO2-enriched exosomes was found to induce proliferation of PAECs by activating their STAT3 pathway in PH [195]. Another study found EV-bound WNT5A signaling protein causing lung fibroblast proliferation in idiopathic lung fibrosis patients [196]. Though there are significant data available on the role of EVs in pulmonary diseases, the involvement of EVs in HTLV-1-associated alveolitis and bronchiectasis still remains unexplored. It is possible that EVs from HTLV-1-infected cells carrying Tax and ICAM-1 [57,82,197] may be a participant in the increased inflammation observed in these patients. A further detailed study is warranted to explore how EVs can contribute in the pathophysiology of pulmonary disease associated with HTLV-1 infection. 

### 3.5. EVs in Uveitis

HTLV-1-associated uveitis (HU) is one of the most common uveitis in endemic areas of Japan [198]. HU is an ocular inflammatory disease caused by intraocular infiltration of HTLV-1-infected CD4^+^ T cells that stimulate the release of inflammatory cytokines (i.e., IL-1α, IL-2, IL-3, IL-6, IL-8, IL-10, TNF-α, IFN-γ, and GM-CSF) [129,199], resulting in immune reactions and inflammation. Patients with HU have painless floaters, blurry vision, and mild retinal vasculitis [198]. Topical or systemic corticosteroids are therapies to manage cytokine production, but a majority of HU patients usually relapse [129,199]. The pathogenicity of HU is characterized by a significant increase in HTLV-1 proviral load [200] that can be correlated with the severity of intraocular inflammation. It has been reported that the proviral load in the HU patients’ eyes was found to be higher than in PBMCs [201]. Additionally, elevated levels of CD4^+^ T lymphocytes, CD4/8 ratio, CD25^+^ T lymphocytes (express IL-2R), as well as decreased CD8^+^ T lymphocytes were observed in HU patients [202], pointing to the importance of these immune cells in HU pathogenesis. 

Additionally, HTLV-1 negatively affects the retinal pigment epithelium (RPE) of the eye which is a monolayer of cells located between the neuroretina and the vascularized choroid. RPE is crucial in maintaining the homeostasis of the outer retina which is considered part of the blood-ocular -barrier (BOB) that protects the eye. RPE expresses various cytokine receptors and their ligands including IL-1, IL-6, IL- 15, TNF, TGF, FGF, IGF, VEGF and PDGF, major histocompatibility complex (MHC) molecules, cell surface adhesion molecules (i.e., ICAM-1), and Fas receptor-Fas ligand (FasL) that are involved in immune and inflammatory responses [203]. ICAM-1 is highly expressed on the surface of RPE cells resulting in an increased permeability and disruption. This in turn increases the susceptibility of RPE cells to HTLV-1 infection [204]. Healthy RPE cells (i.e., uninfected) have anti-inflammatory properties that protect the retina and adjacent RPE cells from being damaged through the inhibition of immune-mediated inflammation. RPE cells release molecules such as TGF-β, somatostatin, thrombospondin and pigment epithelial-derived factor (PEDF) [205]. Some of these factors have been found to stimulate the expression of FasL and IL-10 in monocytes. FasL and IL-10 play roles significant in inactivation or eradication of the activated T cells via induction of apoptosis [206]. 

Even though various studies have explored the pathogenesis of HU, investigating the role of HTLV-1 EVs in HU could potentially paint a clearer picture of the disease and may even provide new therapeutic venues. The role of EVs in regulating immune responses in non-infectious uveitis patients (i.e., autoimmune uveitis) have been investigated in few studies. Human PBMCs from non-infectious uveitis patients exposed to EVs isolated from both unstimulated and cytokines (IL-1β, IFN-γ, and TNF-α)-stimulated RPE cells have been found to block T cell proliferation with no reduction in T cell viability [207]. Furthermore, co-culture of EVs, derived from unstimulated RPE cells, with undifferentiated human monocytes induced cell differentiation toward intermediate monocytes (CD14^+^CD16^+^) as well as the release of TGF-β1 [207]. These findings suggest the possible involvement of EVs secreted by unstimulated RPE in mediating the immunosuppressive properties (via potential inhibition of T cell activation) that are crucial for the healthy eye. However, cytokine-stimulated RPE-derived EVs prompted the release of pro-inflammatory cytokines (TNF-α, IL-6, and IL-8) and monocyte apoptosis [207], thus blocking T cell stimulation by monocytes. 

Additionally, an increase in EV concentration was observed after RPE cells were exposed to an oxidative stressor [208]. These EVs promoted angiogenesis in endothelial cells [208]. Another study showed that EVs obtained from RPE cells treated with an oxidative stressor (i.e., Rotenone) caused a reduction in cell proliferation and survival of normal RPE in a dose-dependent manner [209]. These EVs contained high level of apoptotic protease activating factor 1 (Apaf1) that most likely enhanced the induction of apoptosis and inflammatory response in the normal RPE cells [209]. Lastly, Tax has been found to stimulate the production of reactive oxygen species (ROS) in primary human cells resulting in cellular DNA damage and cellular senescence [210]. EVs containing Tax may be acting as an oxidative stressor-induced ROS in the RPE cells resulting in HU progression. Taken together, these data suggest that EVs may play a critical role in mediating HU pathogenesis. Additional studies are needed to further explore this phenomenon in more depth and detail.

## 4. Treatment Options for HTLV-1-Associated Diseases

Currently, there are no FDA-approved drugs for the treatment of HTLV-1 infection. However, associated disorders can be managed, and antiviral drugs are being repurposed and developed. Reverse transcriptase (RT) inhibitors such as Zidovudine (AZT) and phosphonated carbocyclic 2′-oxa-3′aza nucleosides (PCOANs) have demonstrated consistent inhibition of RT from HTLV-1 isolated from the patients [211]. Other potential therapeutic approaches could be developed based on the targeting of host proteins. Toyoma et al. studied the inhibitory activity of a tetrahydrotetramethylnaphthalene (TMN) derivative, TMNAA, on HTLV-1 [212]. This derivative had a synergistic effect on the inhibition of HTLV-1 when used in combination with the NF-kB inhibitor cepharanthine, making both drugs possible candidates in the treatment of ATLL and other HTLV-1-associated disorders [212]. Vitamin B1 analogue prosultiamine has been shown to decrease the viral load in HAM/TSP patients and induce apoptosis in HTLV-1-infected cells. Urinary symptoms and motor functions have also been reported to improve [213]. The use of monoclonal antibodies to target CCR4^+^ T cells (main cell targets for HTLV-1 infection) in patients with HTLV-1 has also been studied [214]. The antibody treated cells are removed via antibody-dependent cellular cytotoxic effects. For instance, mogamulizumab, an anti-CCR-4-binding monoclonal antibody, was approved to treat ATLL cases in Japan. Based on data obtained from a clinical trial, mogamulizumab could potentially be used to reduce inflammation and neurologic symptoms in patients with HAM/TSP. Use of low doses of mogamulizumab (i.e., 0.003 mg/kg/week) for 21 HAM/TSP patients showed that it is efficient at reducing HTLV-1 proviral load (by 64.9% in 15 days after drug administration) in PBMCs as well as CSF inflammatory indicators (CXCL10 and neopterin) [214]. An improvement in motor ability with a reduction in spasticity was observed in HAM/TSP patients after taking mogamulizumab. In contrast, treatment of ATLL may consists of a higher dose of mogamulizumab (i.e., 1.00 mg/kg/week) [214]. Overall, the drug was deemed to be safe, but some patients experienced side effects including rash, lymphopenia, and leukopenia. Further studies should be conducted to further evaluate the safety of the drug for long-term use especially in terms of the adverse side effects that may arise from suppressing CCR4^+^ T cells [214]. Lastly, the antibody humanized Mik-beta-1 (Hu-Mik-beta1) is an inhibitor of spontaneous lymphoproliferation caused by Tax protein which induces IL-15/IL-15R-α signaling in HAM/TSP patients. A study using ex vivo cells (obtained from HAM/TSP patients) treated with Hu-Mik-beta1 showed a reduction in HTLV-1-specific CD8^+^ T cells [215,216], suggesting less inflammation and damage to the CNS. Hu MiK-Beta1 is currently in clinical trial to evaluate its safety and effectiveness in patients with HAM/TSP [217]. Finally, it is not clear if any of these therapeutic approaches against HTLV-1 also regulate EV release and whether EVs have a role in further enhancing the therapeutic effects of these drugs. 

## 5. Treatments Used to modulate EV Biogenesis, Release, and Uptake

Currently there are no widely accepted inhibitors of EV biogenesis, release (secretion), or uptake, however identification of such drugs may provide valuable options to prevent disease progression. For instance, EV secretion is an important feature of many neoplastic disorders, including ATLL. EVs facilitate tumor growth, immune evasion, tumor angiogenesis, metastasis, and resistance to therapeutic agents by spreading tumor growth factors and modulating immunological tolerance [116]. Tumor-derived EVs are known to be a hallmark of an aggressive disease [218]. Moreover, in the case of virally induced cancers such ATLL, tumor-derived EVs carry viral proteins can possibly upregulate host proteins and exacerbate the course of the disease. EVs may potentially play a major role in HTLV-1 induced cancers as they often contain viral proteins and RNAs that can influence cell cycle in the recipient cells [88,197]. Removal of the tumor-derived EVs from the cell-cell communication environment might provide therapeutic benefits to patients. Despite the various documented roles EVs play in disease progression, there has not been significant progress in the discovery of drugs that can potently inhibit EV biogenesis and secretion. Here, we discuss several drugs that have been screened to inhibit EVs release/uptake.

Current EVs inhibitors can be classified into three categories based on their pharmacological effect on inhibiting EV trafficking (secretion), lipid metabolism (biogenesis), or uptake. Calpeptin, MDL28170, Manumycin A, Tipifarnib, Y-27632, and Neticonazole are drugs known to target EV trafficking, while GW4869, Imipramine, and Pantethine impact the lipid metabolism of molecules that are involved in EV production and secretion [219,220,221]. EV uptake can be inhibited by Dynasore, Annexin V, Diannexin, Cytochalasin B, Cytochalasin D, Filipin, Simvastatin, and proton pump inhibitors (PPIs) [222,223,224,225,226] (Table 2). 

### 5.1. Inhibiting EV Trafficking

It has been observed that cellular activation may change the levels of EVs trafficked and secreted by a cell, modifying the functional effects on recipient cells. EVs may be necessary to maintain homeostasis. For instance, cellular activation has been shown to yield higher levels of EV secretion compared to resting cells. This upregulation in EV trafficking has been observed to occur in parallel with increases in intracellular calcium (Ca^+^) levels and cytoskeletal remodeling [227,228]. Therefore, the control of intracellular calcium levels may allow for the control of EV release. Calpeptin is a known inhibitor of calpain, a Ca^+^-dependent cytosolic cysteine protease. Mechanistically, this drug interrupts the binding of calpain and cortactin (an actin-nucleation factor), restricting MVB formation and protein packaging into vesicles. These events may result in a reduction of EV release (i.e., MVs) [229]. HTLV-1 uses the viral protein p12^I^ to activate the surface protein LFA-1 in T-cells, promoting cell-cell contact and viral spread [230]. Calpeptin may be an option to reduce viral spread since it has been used to inhibit Ca^+^-dependent signaling. HTLV-1 infection via cell-cell contact was downregulated after calpeptin treatment [230]. Another calpain inhibitor, MDL28170, has been shown to slow down EV release [231]. Furthermore, the antibiotic, Manumycin A, has been used in various studies as an EV release inhibitor [232]. Manumycin A is an inhibitor of the Ras farnesyl transferase (i.e., Ras-mediated ERK activation), as well as the neutral sphingomyelinase 2 (n-SMase 2) [232,233]. Both of these molecules are crucial in EV biogenesis (i.e., through ESCRT-dependent/independent pathways) [232,233]. Therefore, blocking their expression results in the reduction of EV secretion. In HTLV-1, Tax may be released in EVs since treatment with Brefeldin A (a virus budding inhibitor) did not affect the presence of tax-associated EVs, but use of Manumycin A on infected cells (i.e., C81) abolished presence of EVs/tax [88]. These data suggest that pharmacological inhibition of EVs is possible in HTLV-1-infected cells, and that this inhibition may also suppress viral cargo important for pathogenesis [88]. Similar to Manumycin A, Tipifarnib is a potent farnesyl transferase inhibitor and has been shown to block EV release from aggressive prostate cancer cells [220]. Tipifarnib is currently in clinical trial phase II for recurrent ATLL (ClinicalTrials.gov Identifier: NCT00082888).

Additionally, Y-27632 is an inhibitor of Rho-associated, coiled coil-containing protein kinase (ROCK). Y-27632 competes with ATP for binding to the kinase which hinders both ROCK1 and ROCK2 enzymes. ROCK induces actin reorganization via membrane deformation, which helps in vesicle formation. Y-27632 blocks EVs shedding from the cell membrane [234]. Neticonazole is also used to inhibit EVs (i.e., exosomes) secretion by reducing the levels of Alix, Rab27a, and nSMase2 [220,221]. A reduction in tumor growth as a result of inducing apoptosis of tumor cells has been observed as well as additional improvement in the survival of colorectal cancer xenograft mouse following neticonazole treatment [221]. The inhibition of EV release by Neticonazole could potentially contribute to cancer treatment regimens (Table 2). 

### 5.2. Inhibiting Lipid Metabolism

EV release can also be inhibited by impairing lipid metabolism. The enzyme nSMase 2 induces the conversion of sphingomyelin to ceramide that is responsible for exosome biogenesis (i.e., controlling the budding of ILVs into MVBs) and the shedding of MV from cells [219,242]. Inhibiting nSMase 2 led to a significant decrease in EV release [219]. The compound, GW4869, has been extensively used in numerous studies to inhibit nSMase 2, resulting in the depletion of ceramide formation and the subsequent release of exosomes and MVs. Cambinol is considered a potent inhibitor of nSMase2 due to its unique properties (i.e., “higher aqueous solubility and lower molecular weight”) [237,243]. The amount of EV production has been shown to decrease in human endometrial adenocarcinoma cells post-Cambinol treatment [237]. 

Imipramine, in combination with other drugs, has been used as an antidepressant and neurogenic bladder therapy for HAM/TSP patients [15,166]. Imipramine is also an inhibitor of acidic sphingomyelinases (aSMase) that is able to block EV release [238]. Upon activation of the ATP receptor P2X7, aSMase is transferred into the outer leaflet of the plasma membrane in order to generate ceramide from sphingomyelin for MV biogenesis [219]. Imipramine induces the degradation of aSMase that later detaches from the membrane, blocking MV shedding [219]. Osteoblasts treated with Imipramine have shown a reduction in EV secretion [238] (Table 2). Another EV release inhibitor, Pantethine is formed from pantothenic acid and is involved in lipid metabolism (i.e., inhibits both total fatty acids and cholesterol synthesis) [239]. Depletion of lipids in the cell alters membrane fluidity and MV biogenesis by limiting the presence of phosphatidylserine on the outer leaflet of the plasma membrane [239,240] (Table 2). These data provide evidence of the use of Imipramine, or Pantethine, as potential selective inhibitors of MVs.

Due to the heterogeneity of EV subpopulations (exosomes, MVs, and apoptotic bodies), the inhibition of different pathways for EV biogenesis and release may be achieved by using a combination of some of the previously described inhibitors. Knowing which EV subpopulation is mainly responsible for disease development would be significant in pinpointing which of the EV type should be blocked from release. In this case, cell viability and proliferation should be measured to confirm that changes in EV quantities are related to the action of an inhibitor, and not to drug toxicity. Since EVs may play a role in the viral life cycle and in the associated disease progression, inhibition of EV release becomes an attractive option. The arsenal of EV release inhibitors is increasing over time and there are more drugs discovered to inhibit this process. 

### 5.3. Inhibiting EV Uptake

EVs have numerous surface molecules such as proteins and glycoproteins that may bind many cell receptors, making possible the interaction between cells and exchange of cargo [222]. Normal cellular functions, such as phagocytosis in many immune cell types, allow EV uptake or direct fusion with the plasma membrane (i.e., clathrin- or caveolin-mediated endocytosis, macropinocytosis, and lipid raft-mediated internalization) [222]. In order to track EV uptake by recipient cells, EVs can be labeled with either fluorescence (e.g., dye lipid membrane, PKH67, and PKH26) or GFP which tags EV-associated proteins including tetraspanins CD9 and CD63 [222]. These EV uptake labeling methods could be useful to evaluate how inhibitors or blocking antibodies act on the EV uptake process. 

Various EV subpopulations may use multiple routes of entry into target recipient cells and may differ depending on the cell type. In regard to HTLV-1 EVs, the molecular mechanisms of uptake by recipient cells has not been extensively studied. EV dose, incubation time, pH, and temperature (37 °C vs. 4 °C) are factors that could be evaluated in order to shed light on the methods of EV uptake. Since EVs have been shown to mediate disease progression, preventing their uptake into recipient cells using different drugs or compounds could be a beneficial strategy. For instance, Dynasore is an inhibitor of Dynamin-2 that regulates EV uptake via clathrin- and caveolin-dependent endocytosis [222]. It has been shown the use of dynasore in co-culture of HTLV-1-infected T-cells and immature DCs (human monocytes-derived dendritic cells) resulted in a reduction in viral entry [244]. This finding suggests that dynamin-mediated endocytosis regulates HTLV-1 entry in immature DCs. Therefore, such an inhibitor could be used to limit viral spread. Annexin V or Diannexin has been used to target phosphatidylserine on the surface of cancer cell-derived EVs (i.e., MV) resulting in blocking their entry into recipient cells [223]. Cytochalasin B and Cytochalasin D are actin polymerization inhibitors that have been used to inhibit EV uptake via endocytosis processes that necessitate cytoskeletal remodeling [224,225]. The EV uptake via lipid raft-mediated endocytosis can be blocked by Filipin or Simvastatin [222]. Simvastatin has a dose dependent effect on inhibition of small GTPases, affecting cellular processes, such as proliferation and apoptosis, resulting in decreased viability of ATLL cells [245]. To impede membrane fusion, proton pump inhibitors (PPIs) have been utilized to change the pH condition of cells thereby suppressing the entry of EVs (i.e., exosomes) into melanoma cells [222,226]. In order to use EVs for therapeutic intervention, future studies should focus on EV cell targeting, modes of uptake, and especially the routes of intracellular trafficking in recipient cells.

### 5.4. Current Challenges to EV inhibition

There are many challenges of EV inhibitor-based therapeutics mainly due to the lack of understanding of EV biogenesis, and lack of in vivo studies. The optimal strategy to prevent the functional effects of an EV is to stop its biogenesis. However, we do not fully understand how to efficiently isolate and identify individual populations of EVs generated by the multiple EV biogenesis pathways (e.g., ESCRT-dependent, -independent, among others). This is a current challenge faced by the entire EV field, where EV-associated proteins are being used as potential biomarkers to determine their origin. In HTLV-1, the overarching issues related to pharmacological inhibition of EVs are lack of understanding of the EV subpopulation that is targeted by a drug, the side effects, and the EV type responsible for pathogenesis. For example, the use of an EV inhibitor may have effects on certain EV subpopulations, while leaving others functional. It is important to first identify which EV type is being inhibited by a particular drug prior to use in EV therapeutic strategies. Additionally, the impact of these EV inhibitors on EV biogenesis/secretion from normal cells should be meticulously investigated. Distinguishing between non-pathogenic and potentially pathogenic EVs (derived from infected cells) based on specific cargo (e.g., viral RNAs/proteins) would be challenging. However, identifying these EV populations would offer significant benefits for controlling disease progression. A combination of multiple drug types may be necessary to fully inhibit EV production. Albeit the complete shutdown of EV production could also result in undesired side effects related to deregulation of processes that depend on background levels of EV production. For instance, some side effects of the EV release inhibitor, Imipramine, may include blood disorders, suppression of the immune response, nausea, vomiting, and other complications [219]. Overall, identifying the EV subpopulations potentially responsible for pathogenesis, while targeting shutdown of specific EV production pathways may allow for effective suppression of disease while mitigating side effects from broad inhibition. Instead of broadly inhibiting EV biogenesis, which may yield varied side effects, a two-pronged approach to first inhibit biogenesis of specific EV populations (i.e., pathogenic), followed by inhibition of EV uptake by the target cell, may provide for a more effective strategy with reduced side effects.

## 6. Strategies for Preventing the Viral Transmission

Effective methods to control the transmission of HTLV-1 infection should be carefully considered in highly epidemic areas. In relapse cases, treatment options for HTLV-1-associated diseases are not as effective. Moreover, there is limited education and awareness of HTLV-1 and no available vaccinations, thereby making transmission one of the most significant contributors of the high prevalence observed worldwide. To tackle these issues, we must first develop effective modes of detecting the virus versus EVs and follow up with preventive and supportive strategies for the populations at risk. Most of nucleic acid or protein-based detection methods may typically analyze viral proteins and particles to diagnose and decide on a treatment course. However, the EVs in these samples may be informative and equally important. Current blood collection and techniques used to separate coagulation factors away from the plasma and subsequently isolate white blood cells discard the supernatant material that could be clinically relevant in the near future. Therefore, it is possible that the plasma used for diagnostic of HTLV-1 infection also carries EVs. Recent evidence has shown that the supernatant from HTLV-1-infected cells contains a mixture of EVs (packaged densely with viral proteins and RNA) and potential non-infectious viral particles [57]. EV subtypes may be of similar diameter to the HTLV-1 virion and carry most viral proteins that comprise the virion. These EVs isolated from the CSF of patients are still able to elicit immune responses, such as activation of Tax-specific cytotoxic T-cell lysis [82], and are able to induce cell-cell contact and increased viral spread in cell lines and PBMCs [57]. Similarly, we recently observed that HIV-1 in PBMCs may modulate packaging of viral proteins and inflammatory cytokines into EVs, depending on the transcriptional status of the virus. These resulting EVs were able to cause inflammation in HLM-1 indicator-indicator recipient cells, as evidenced by the release of TNF-α and viral activation [246]. These data reveal the importance to clarify blood and plasma samples from EVs and to consider EVs as a biomarker of disease severity, in particular because of the potential EV-mediate effects reported that may contribute to disease severity.

To detect and identify regions of the world with increasing trends of transmission, universal screening requirements are necessary, especially in cases of blood donations/transfusions, prenatal care, emergency medicine, drug abuse, and during routine screening for sexually transmitted infections (STIs). These are the high-risk groups that ought to be targeted in order to curb further spread of the virus and distinguish between infections virions and EVs. Few countries including the United States, Canada, Western European countries (e.g., UK, France, Demark, Greece, Portugal, Romania and Sweden), and Japan have applied routine screenings for HTLV-1 among blood donors [247]. Unfortunately, it has not been implemented in many countries that are highly HTLV-1 endemic, such as Gabon [34]. Preventative strategies should also include a plan to educate seropositive patients and other high-risk individuals in care, treatment options and prevention of viral transmissions, such as in the cases of mother to child, between spouses, and between drug users who share needles. Special attention must be given to providing support for HTLV-1-positive mothers during prenatal and postnatal phases to prevent HTLV-1 transmission to newborns. It is necessary to have a program that would facilitate alternatives to breastfeeding, so mothers of low-income or in isolated communities (i.e., Australian aboriginals) have a free source of nutrition for their child. By providing alternative food sources (i.e., baby formula), the dependence on breastfeeding, and the risk of MTC viral transmission can be reduced. This kind of control could prevent children born to seropositive HTLV-1 mothers from getting ATLL or HAM/TSP later in life. 

## 7. Conclusions and Future Perspectives

Even though it was discovered over 30 years ago and despite its association with debilitating diseases, namely ATLL and HAM/TSP, HTLV-1 has remained relatively neglected by public health officials. Although approximately 5–10 million people are believed to be infected with HTLV-1, its global prevalence may be much higher than is currently estimated [1,2,3,4,5]. This is due, in part, to pockets of highly endemic regions where it is often difficult to obtain reliable epidemiological data [6]. Thus, it is critical to pursue research that will not only further our understanding of HTLV-1 pathogenesis but also contribute to the development of novel diagnostic or therapeutic strategies.

The current knowledge surrounding EV-associated functions, especially in the context of cancer and HIV-1, could also be applicable to HTLV-1 EVs. Along these lines, future research should focus on the effects of HTLV-1 EVs on cellular transformation. Studies of this nature could provide valuable insight into the mechanisms contributing to viral spread, modulation of the host immune response, and disease progression. For example, in certain cancers, it has been shown that the EV-associated integrin αvβ3 can reprogram recipient cells and promote a malignant phenotype [248,249]. Moreover, proteomic profiling of EVs derived from cancer cells revealed that the levels of integrin (e.g., α6, αv, and β1) directly correlate with severity of tumor in epithelial cancers [250]. There is also evidence to suggest that human papillomavirus (HPV) oncoprotein expression can alter the miRNA profiles of EVs and that EV-enriched miRNAs may contribute to inhibition of apoptosis and necrosis in recipient cells [251].

Future experiments should also aim to better define the biochemical and functional characteristics of different HTLV-1 EV subtypes. This information could be useful for determining whether certain subpopulations of EVs from HTLV-1-infected cells may be more toxic than others. To illustrate this point, a comparative analysis of the pathological proteins associated with EVs from amyotrophic lateral sclerosis (ALS) patients found that superoxide dismutase 1 (SOD1) was more enriched in exosomes, while TAR DNA-binding protein 43 (TDP-43) and the RNA-binding protein Fused in Sarcoma (FUS) were more concentrated in microvesicles. Interestingly, this same study found that the mean size of both exosomes and microvesicles from ALS patients was larger than those from healthy controls [252]. Therefore, the proteomic analysis and RNA sequencing of HTLV-1 EV subtypes has the potential to not only identify key functional cargo but to also identify EV-associated molecules that could serve as biomarkers for the progression of HTLV-1 and its related pathologies. Overall, this information could assist in the development of novel strategies aimed to limit the spread of HTLV-1.

## Figures and Tables

**Table 1:** The geographic distribution of HTLV-1 and its prevalence rates.

HTLV-1 Prevalence Rates			
Continent	Country/Region	Seroprevalence Rates	References
Australia	Certain Indigenous populations of Queensland	>40%	[26,27]
Asia	Southwestern isles of Japan including Shikoku, Kyushu, and Okinawa	37%	[28]
	Taiwan	0.1–1.0%	
	The Mashhad area of northeastern Iran	up to 3%	[4,29]
	China, Iraq, Israel, Lebanon, Saudi Arabia, Turkey, Singapore, South Korea	<0.03%	[7,30,31]
Africa	Morocco	0.6%	[9,32]
	Benin, Cameroon, and Guinea-Bissau	>5%	
	Côte d’lvoire	0.5–2%	[6]
	Burkina Faso, Chad and Gambia	1–1.2%	
	Senegal	0.2–1.2%	[33]
	Togo	1–1.6%	
	Kenyan	2.7–19.5%	
	Congo	3.2%	
	Nigeria	5.5%	
	Mozambique	1.5%	
	Guinea	1.05%	
	Ghana	0.5–4.2%	
	Malawi	0.63%	
	Seychelles	>15%	
	Gabon (rural adult Gabonese populations)	8.7%.	[7,34]
	Central African Republic	0.6%	[6]
	South Africa	1%	[33]
Europe	France	0.0039%	[9,35]
	The United Kingdom	0.03%	[36]
North America	United States (especially in New York, NY, and Miami, FL)	0.035%	[9,37]
	Caribbean islands (Jamaica)	5%	
South and Central America	Chile	0.73%,	[9,38]
	Argentina	0.07%	[9,38]
	Colombia, Venezuela, Guyana, Surinam, Panama, and Honduras	5–14%	[39]
	Brazil (in Bahia)	>15%	[40]

**Table 2:** Regulators of EV biogenesis/release and uptake from cells.

Mechanisms of Action	Drugs	Effect	Block	References
EV trafficking/EV release	Calpeptin MDL28170	Inhibits calpain, cleavage of cortactin, and MVB formation	EV biogenesis/release	[229,231]
	Manumycin	Blocks farnesyl transferase, hinders Ras from binding to plasma membrane, prevents budding from plasma membrane, stimulates n-SMase 2 activity	EV release	[232,233]
	Tipifarnib	Inhibits the activity of farnesyltransferase	EV release	[220]
	Y27632	Inhibits Rho-associated kinase ROCK1 and ROCK2	EV release	[234]
	Neticonazole	Decreases Alix, Rab27a, and nSMase2	EV release	[220,221]
Lipid metabolism/EV release	GW4869	Inhibits nSMase 2 and subsequent incorporation of ceramide into EVs	EV release	[235,236]
	Cambinol	Inhibits nSMase2	EV release	[237]
	Imipramine	Promotes the degradation of aSMase that later detaches from the plasma membrane	EV (i.e., MV) release	[219,238]
	Pantethine	Inhibits fatty acid and cholesterol synthesis to block MV biogenesis	EV (i.e., MV) biogenesis	[239,240]
EV uptake	Dynasore	Blocks Dynamin-2-mediated clathrin- and caveolin-dependent endocytosis	EV uptake	[222]
	Annexin V Diannexin	Blocks EV ligand (e.g., phosphatidylserine EV surface)	EV uptake	[223,241]
	Cytochalasin B Cytochalasin D	Blocks actin polymerization and endocytosis	EV uptake	[224,225]
	Filipin Simvastatin	Blocks EV uptake via lipid raft-mediated endocytosis	EV uptake	[222]
	PPIs	Alters pH and blocks EV uptake via membrane fusion	EV uptake	[222,226]
